# A metadata approach to evaluate the state of ocean knowledge: Strengths, limitations, and application to Mexico

**DOI:** 10.1371/journal.pone.0216723

**Published:** 2019-06-12

**Authors:** Juliano Palacios-Abrantes, Andrés M. Cisneros-Montemayor, Miguel A. Cisneros-Mata, Laura Rodríguez, Francisco Arreguín-Sánchez, Verónica Aguilar, Santiago Domínguez-Sánchez, Stuart Fulton, Raquel López-Sagástegui, Héctor Reyes-Bonilla, Rocío Rivera-Campos, Silvia Salas, Nuno Simoes, William W. L. Cheung

**Affiliations:** 1 Institute for the Oceans and Fisheries, The University of British Columbia, Vancouver, British Columbia, Canada; 2 Instituto Nacional de Pesca y Acuacultura, Guaymas, Sonora, México; 3 Environmental Defense Fund de México, La Paz, Baja California Sur, México; 4 Instituto Politécnico Nacional, Centro Interdisciplinario de Ciencias Marinas, La Paz, Baja California Sur, México; 5 Comisión Nacional para el Conocimiento y Uso de la Biodiversidad, Ciudad de México, México; 6 University of California San Diego, Scripps Institution of Oceanography, La Jolla, California, United States of America; 7 Comunidad y Biodiversidad, Cancún, Quintana Roo, México; 8 Universidad Autónoma de Baja California Sur, La Paz, Baja California Sur, México; 9 SmartFish Rescate de Valor, A.C., La Paz, Baja California Sur, México; 10 Instituto Politécnico Nacional, Centro de Investigación y Estudios Avanzados del Instituto Politécnico Nacional, Mérida, Yucatán, México; 11 Universidad Marista de Mérida, Mérida, Yucatán, México; 12 Universidad Nacional Autónoma de México, Unidad Multidisciplinaria de Docencia e Investigación – Sisal, Yucatán, México; 13 Laboratorio Nacional de Resiliencia Costera, Laboratorios Nacionales, Ciudad de México, México; 14 Texas A&M University, Corpus Christi, Texas, United States of America; Swedish University of Agricultural Sciences and Swedish Institute for the Marine Environment, University of Gothenburg, SWEDEN

## Abstract

Climate change, mismanaged resource extraction, and pollution are reshaping global marine ecosystems with direct consequences on human societies. Sustainable ocean development requires knowledge and data across disciplines, scales and knowledge types. Although several disciplines are generating large amounts of data on marine socio-ecological systems, such information is often underutilized due to fragmentation across institutions or stakeholders, limited standardization across scale, time or disciplines, and the fact that information is often not searchable within existing databases. Compiling metadata, the information which describes existing sets of data, is an effective tool that can address these challenges, particularly when metadata corresponding to multiple datasets can be combined to integrate, organize and classify multidisciplinary data. Here, using Mexico as a case study, we describe the compilation and analysis of a metadatabase of ocean knowledge that aims to improve access to information, facilitate multidisciplinary data sharing and integration, and foster collaboration among stakeholders. We also evaluate the knowledge trends and gaps for informing ocean management. Analysis of the metadatabase highlights that past and current research in Mexico focuses strongly on ecology and fisheries, with biological data more consistent over time and space compared to data on human dimensions. Regional imbalances in available information were also evident, with most available information corresponding to the Gulf of California, Campeche Bank and Caribbean and less available for the central and south Pacific and the western Gulf of Mexico. Despite existing knowledge gaps in Mexico and elsewhere, we argue that systematic efforts such as this can often reveal an abundance of information for decision-makers to develop policies that meet key commitments on ocean sustainability. Surmounting current cross-scale social and ecological challenges for sustainability requires transdisciplinary approaches. Metadatabases are critical tools to make efficient use of existing data, highlight and address strengths and deficiencies, and develop scenarios to inform policies for managing complex marine social-ecological systems.

## Introduction

The ocean contributes to human wellbeing by providing a diversity of goods and services such as food, energy, transport, among others as well as a source of cultural and recreational values to people [1,2]. However, drivers from human activities, including climate change, excessive extraction of marine resources, and pollution are impacting global marine biodiversity and ecosystem services [3–6] and causing undesired social and economic outcomes [7]. Mitigating and managing these human drivers, and achieving sustainable ocean development, requires data from different disciplines, that spans longest time ranges possible, and that covers different geographic scales. Only with this diverse and complementary knowledge can policymakers evaluate status and trends, and set clear targets, for effective policy design and implementation [8]. Adopting a multidisciplinary approach has been recently recognized in partnerships aiming to achieve the cross-disciplinary United Nations (UN) Sustainable Development Goals [9]. Yet despite a call for global shift towards open science and the benefits imbedded [10], data identification, access, and sharing continue to be a challenge throughout the world [11].

Metadata is important in the harmonization of existing data across scales, disciplines and domains. Metadata refers to the information required to understand the data such as the data type, content, source, quality, format, structure, and accessibility [10,12]. Metadata repositories (and their development itself) can assist in addressing the challenges of data sharing, by improving data access, fostering collaboration among stakeholders, and facilitating subsequent analyses and data refinement [13,14]. Various research fields related to socio-ecological marine systems have generated large amounts of data. However, such information is often underutilized because it is scattered and held by different institutions or stakeholders, not standardized, and either not readily found nor widely accessible [6,8,15]. Metadata is particularly useful for developing nations with limited research capacity [11] and where data exist but are perceived to be limited or unavailable [16].

Country level repositories for marine systems including metadata have been created, with examples including Australia [17], Canada [13], and the Canary Islands in Spain [18]. The Integrated Marine Observing System (IMOS) is an Australian national collaborative research project that includes a metadatabase allowing users to see dynamic graphs, enter metadata, and access data [17]. Such database resulted in hundreds of peer review publications, book chapters and reports [19]. In Canada [13], a metadata repository was created with the objective of identifying thematic and information gaps in marine research for the Arctic, Pacific, and Atlantic regions, and was subsequently used to evaluate national policy progress towards the Convention on Biological Diversity—Aichi Targets (CBD) [14]. The Integrated Marine Data Repository of the Canary Islands (REDMIC) includes data, metadata, research documents, maps, and interactive graphs related to the marine environment, which have supported regional decision making and research [18]. All of these initiatives aim to increase data access, support metadata research, and improve science-based decision making related to marine environmental policies.

In this study, we develop a framework for interdisciplinary metadatabase of marine systems, with the aims of assessing existing research and information status and trends to support decision making for sustainable ocean development. We applied this framework to Mexico as an example of a developing nation with extensive marine and coastal areas [15]. As in other parts of the world, multiple academic (e.g. research institutions [20]), government [21], civil society organizations (CSO) [22], and private organizations and institutions generate and host a wealth of data from multiple research fields. However, information on these data—and the data itself—is not always visible, accessible, or searchable in a standardized format, so that individuals working in specific fields may be unaware of past or current related research. Further, the full scope of research—both temporally and spatially—is not easily available to policymakers. These limitations can be addressed through a dedicated effort centered around building and maintaining a metadata repository.

This study describes the processes of metadatabase design, compilation, and methods to link and harmonize datasets from different scales and domains; we then offer examples of metadata-based analyses of historical, regional, and thematic trends. Creating and maintaining an open-source metadata repository can facilitate interpretation of information through public consultation and data sharing. Metadata analyses are critical to help identify data gaps and promote networking and collaboration among a wide array of individuals, institutions and organizations.

## Materials and methods

To develop a metadatabase of ocean research in Mexico (hereafter referred to as the MDB) we framed a four-stage process: (1) development of the MDB structure; (2) identification, outreach and compilation of available repositories and datasets; (3) development of protocols for metadata inclusion and sharing [13]; (4) publication of the MDB in an accessible, open source and long-term stable platform with a partner institution (The National Commission for Biodiveristy, CONABIO [23]). We then provided examples of meta-analyses for identification of information trends and gaps. The final MDB can be found at https://www.infoceanos.conabio.gob.mx.

### Metadata structure

**Fig 1:**
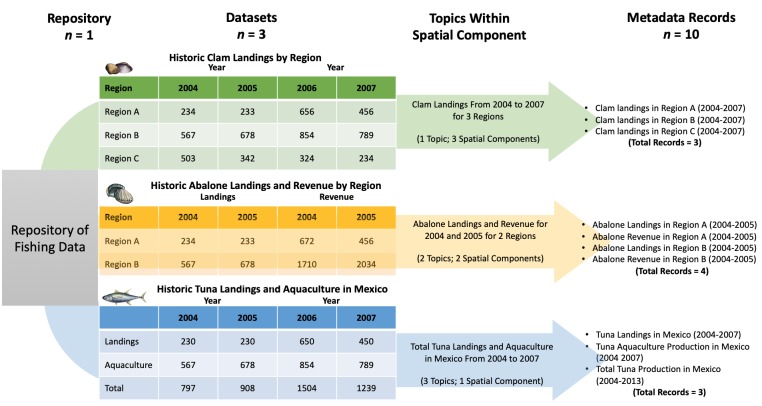
A schematic diagram of the metadata compilation process. From the original repository, three different datasets are represented: the first dataset contains one topic: “landings”, the second contains two topics: “landings” and “revenue”, and the third contains three topics: “landings, “aquaculture”, and “totals”. In addition, each dataset has multiple spatial components. The last column shows how the records would appear in the metadatabase.

There are five hierarchical levels to the MDB structure: Metadatabase > Repository > Dataset > Record > Data point (Fig 1). The metadatabase includes the metadata of datasets, while repositories are structures that compile multiple datasets. Repositories can exist as web-based data sources (e.g. Ocean Biogeographic Information System (OBIS) [24]), thematic reports that contain data (e.g. Mexican Official Catch Statistics [25]), or as institutional, laboratory or research project encompassing multiple datasets (e.g. the species catalogue of the National University’s Institute for Marine Science and Limnology, UNAM-ICMyL [26]). Metadata records are individual entries that describe each dataset within a repository (e.g. ‘clam landings in region A’, or ‘clam landings in region B’; Fig 1). Metadata records contain descriptions of existing data, but not the data themselves; in marine metadatabases these descriptions may include information about fisheries landings, species distributions, or fuel cost of fishing. A data point is a single item of information within a record. For example, a metadata record of annual fish (species specific) population abundance data from 2000 to 2003 includes four (yearly average) data points of estimated abundance data. Records are scale-specific spatially; for example, fisheries catch can be recorded by regional level or country level.

### Metadata categories

Standardization of information within a metadatabase structure provides guidance for consistent description of new data subjects (e.g. abalone, clam, tuna) and types (e.g. methods, units of measurement, and details of experimental design) [12,17,27]. Here, we assigned metadata fields (information categories) to maximize flexibility to accommodate multi-disciplinary data and allowing for various meta-analyses. Initially, the structure was adapted from a previous metadatabase developed for Canadian oceans [13], with subsequent modifications (mainly to ensure compatibility of geographical and species nomenclature with existing frameworks in Mexico) following suggestions in meetings with ocean experts as described in the following section on metadata collection. The key difference between the structure of the MDB and the previous effort for Canada is that the metadata records in the latter represent a particular repository of information (e.g. a report or a database), with a metadata field indicating the number of unique time series within the record. In the MDB, each time series is a unique metadata record and a field notes its corresponding repository. While this structure requires somewhat more effort to input each time series individually, the resulting metadatabase is easier to analyze and allows for more specific information to be added to each record if necessary. The final MDB structure includes 29 categories ranging from general information (e.g. region or subject) to specific metadata including number of data points in the dataset and corresponding research fields (S1 Table).

### Metadata collection

**Fig 2:**
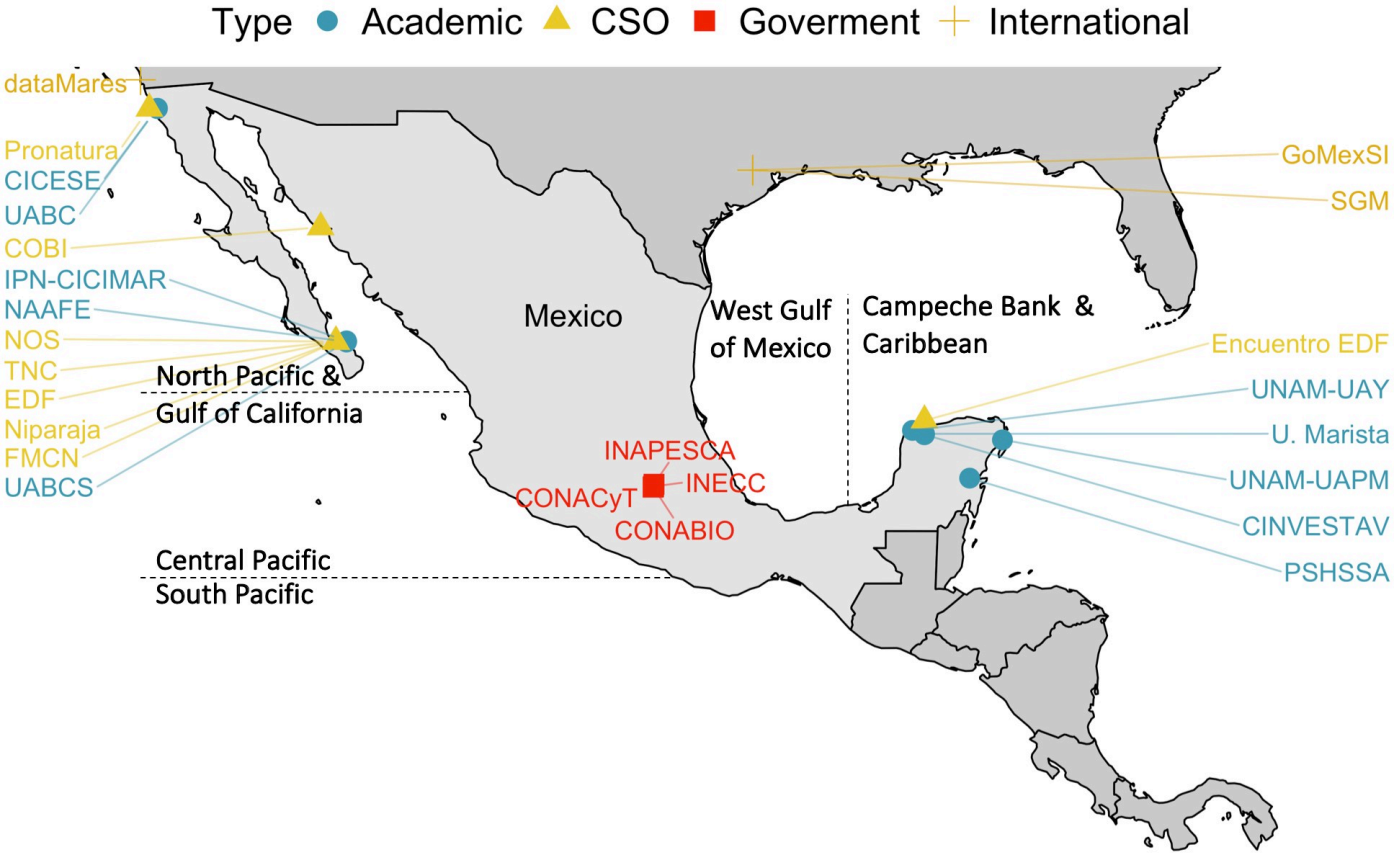
Locations where metadata workshops were held and contributing institutions. Abbreviations in S3 Table. Map reprinted from Natural Earth (naturalearthdata.com).

Compilation of metadata began with a review of public online repositories including OBIS [28] and the UN’s Food and Agriculture Organization (UN-FAO) fisheries statistics [29], followed by federal government catalogues such as the Mexico’s Fisheries and Aquaculture Yearbook [25], and datasets produced and hosted by universities and CSOs working with the marine environment. Using the first MDB developed with public data as a platform for discussion, we held a series of 20 workshops (~30 people each) with research groups (including universities, government researchers and CSO) in eight cities throughout Mexico regions (Fig 2). This was followed-up by in-person and virtual meetings, as well as presentations at national and international conferences to highlight progress and encourage others to contribute and collaborate (S2 Table). We additionally meet with four Mexican federal governmental institutions (CONACyT- National Council of Science and Technology [30], INAPESCA-National Institute of Fisheries and Aquaculture [31], INECC-Ecology and Climate Change Institute [32], and CONABIO [23]), and well-established data repository initiatives (e.g. dataMares [33], FMCN-Monitoreo Noroeste [34]) to include their data in the metadatabase. While this represents an important first effort, it does not comprise all the potential data sources in Mexico highlighting the importance of continuing the current effort.

### Types of data sources

We included all available data sources in the MDB. Firstly, we attempted to include all available data related to Mexican ocean that were publicly available through the internet. These include data from academic, environmental CSO, governmental, international, and private (e.g. industry or personal non-academic) institute and organizations. Another source was unpublished data that were directly kept and maintained by stakeholders and/or institutions. The followings summarize some of the institutions that contributed data to the MDB.

#### a. Academia

Academic data sources include any database hosted by a public or private academic institution in Mexico. Sources with comparatively large available data include the Digital Climatic Atlas of Mexico hosted by the National University (UNAM) [35] which has an extensive open-access compilation of datasets on physicochemical parameters used in, among other uses, climate change models. The UNAM’s academic unit in Sisal, Yucatán (UNAM-UAY) provided information on topics including oceanographic, ecological, fisheries, biological, and tourism data [36]. Finally, The Center for Research and Advanced Studies of the National Polytechnic Institute (CINVESTAV-IPN) holds extensive information on fisheries and tourism, mainly in the Yucatan peninsula [37].

#### b. Governmental institutes

Through a 2015 Mexican decree that establishes regulations for open data, the Mexican federal government made an unprecedented effort to host and make available thousands of public datasets through a national Open Data Portal [38,39]. While the site does not comprise all information generated through decades of public programs, it represents a source of more than 500 datasets related to corruption, economic development, public services, climate change and human rights [39]. These types of data, although not uniquely related to marine ecosystems, are nonetheless important in considering many aspects of socio-ecological interactions that do indeed matter for ocean policy design [8]. In addition to what can be found in the portal, governmental agencies also have data on their institutional web sites. Among the largest repositories in the metadata set are the Secretariat of Economy [40], the fisheries commission CONAPESCA [25], and CONABIO [41]. All data from these and other institutions featured in the metadatabase are public and immediately available at the moment of consultation through reports, internet portals, and yearbooks.

#### c. Civil society organizations (CSO’s)

CSOs are sources of information that include fisheries, conservation, oceanography and sociological data. Comunidad y Biodiversidad, A.C (COBI) contributed the largest CSO repository in the metadatabase. This CSO aims to preserve marine ecosystems that are deteriorating due to unsustainable exploitation of natural resources and has extensive monitoring programs dating back over two decades [22]. FMCN-Monitoreo Noroeste project is the second largest source of metadata from CSOs in the MDB and is itself a repository for monitoring data (~1,000 datasets) including efforts from 20 CSOs [34].

#### d. International academic sources

International research groups hold a variety of data for Mexico specifically at the global scale. dataMares and OBIS are the main international repositories available in the MDB. dataMares is an open source platform based at the University of California, San Diego, that hosts and facilitate access to robust scientific data related to Mexican coasts [33]. OBIS is a global open-access data and information repository on marine biodiversity [24]. In addition, the Arizona-Sonora Desert Museum has an extensive checklist of invertebrates of the Gulf of California, the University of British Columbia through the Changing Ocean Research Unit [42] and Fisheries Economic Research Unit [43], holds more than three thousand records on fisheries economics, model projections on climate change and the associated changes in biodiversity and fisheries catches. Lastly, FishBase [44] and SeaLifeBase [45], online databases of marine life, provide life history data, trophic ecology, and other issues for more than two thousand species occurring in Mexico.

### Metadatabase analysis

The MDB analysis was performed using the statistical software R-Studio (R) Version 1.1.463 with the packages data.table [46] and tidyverse [47]. We compared different metadata categories by number and percentage of records available by research field. Analyses include spatial and temporal distribution of the metadata collected, the amount of metadata collected by taxa, research field, and type of data source, as well as the socio-ecological relationship of the metadata. All figures were produced using the R packages ggplot2 [48], cowplot [49], ggpubr [50], ggrepel [51], gridExtra [52] and wesanderson [53].

For the spatial component we used the packages ggplot2 [48] and sf [54], and Mexico’s shapefile was made with Natural Earth data (http://naturalearthdata.com). Although other spatial divisions exist for Mexico (e.g. CONABIO identifies five marine ecoregions, CONAPESCA identifies six fishing regions), we had to standardize the spatial division in order to include multidisciplinary data (Fig 2). In addition, “Subject names” such as “shrimp”, “shrimps”, “shrimp without head” were standardized as “Shrimp”, and scientific names were updated and corrected for typos with the package taxize [55].

To identify thematic trends, we counted the number of records in the metadatabase, as well as the amount of data points (years of data) available in each record for the years of collection. All metadata was categorized based on their socio-ecological interaction using the DPSIR (Drivers, Pressures, State, Impacts, and Response) framework [56]. Accordingly, Benefits represent social benefits from natural systems (e.g. fisheries landings), Pressure (which we here equate with *Drivers*) represents any pressure from human activities to nature (e.g. fishing effort), Response considers actions that reduce pressure on natural systems (e.g. limiting fishing effort), finally State refers to the status of natural systems (e.g. stock assessments). We used the package networkD3 [57] to analyze the relation between records, institutions, research topics and DPSIR. Finally, we ran Chi-Square Test of statistical difference [58] in the number of records between each variable to describe significant differences.

It is possible that some records include duplicated datasets. We used R to automatize the identification of redundant sources of information (e.g. institutions with the same database). In addition, when possible, we asked data owners and repository curators if a database was already published in another repository. However, given the size of the metadatabase and extensive efforts to identify duplicated records, we do not expect this to be a significant issue. Records representing the same dataset (e.g. CONAPESCA catches and dataMares catches) but with different levels of processing (e.g. cleaned-up data or different years) were kept as separate records in the MDB.

## Results

**Fig 3:**
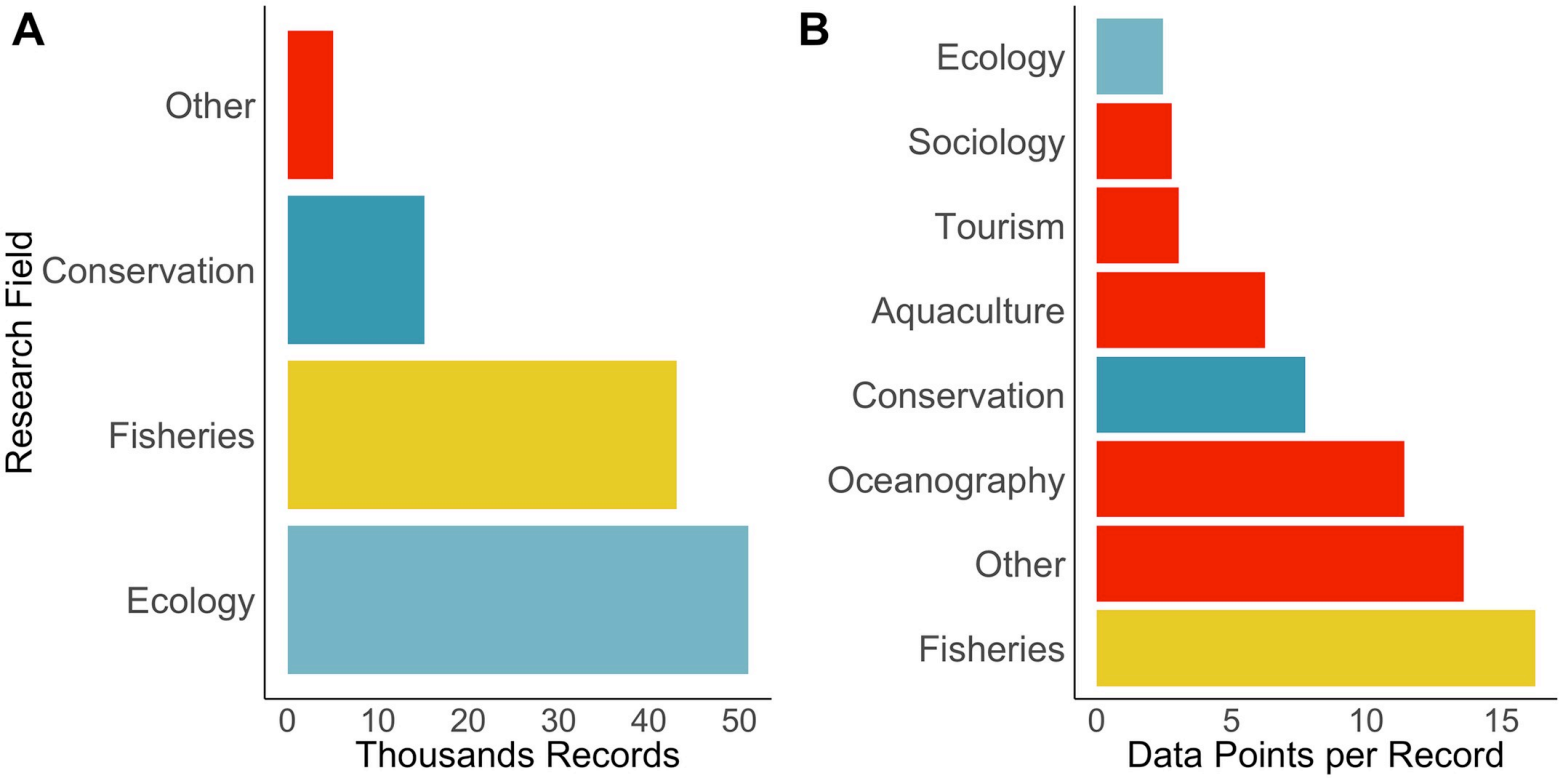
Number of records per research field. A: Thousands of Records. B: Data points per records. Category Other in A represents all of the color-matching categories in B. Category Other in B represents mainly shipping.

As of October of 2018, the metadatabase of marine research in Mexico currently includes 114237 records, from datasets contained in 216 repositories held by academic (n = 19), governmental agencies (n = 22), inter-governmental (n = 2), CSO (n = 21), and international data sources (n = 29). Records are not equally distributed across research fields ($X^{2}=337060$, d.f. = 10, *p* < 0.001), with Ecology comprising 45% of all records, followed by Fisheries with 38% (Fig 3).

International sources (e.g. Global Biodiversity Information Facility-GBIF; dataMares) contributed the highest number of records for Mexico (49%), though these include data collected by Mexican researchers, in Mexican institutions, or funded by the Mexican government [59,60]. In general, metadata records are dominated by academic sources (across multiple topics) and government sources (mainly “Fisheries”) sources. While data sources varied among types of institutions, dataMares (52 datasets mostly on “Fisheries” representing more than 22,000 metadata records), Datos Abiertos Mx (90 datasets from nine different government agencies), and OBIS (19,000 records for more than 13,000 species) represent 46% of all records. Only 20 datasets are classified as private within the metadata (“Dataset Available” category), suggesting that virtually all data here analyzed are open access and available for consultation, and authors likely open for collaborations.

**Fig 4:**
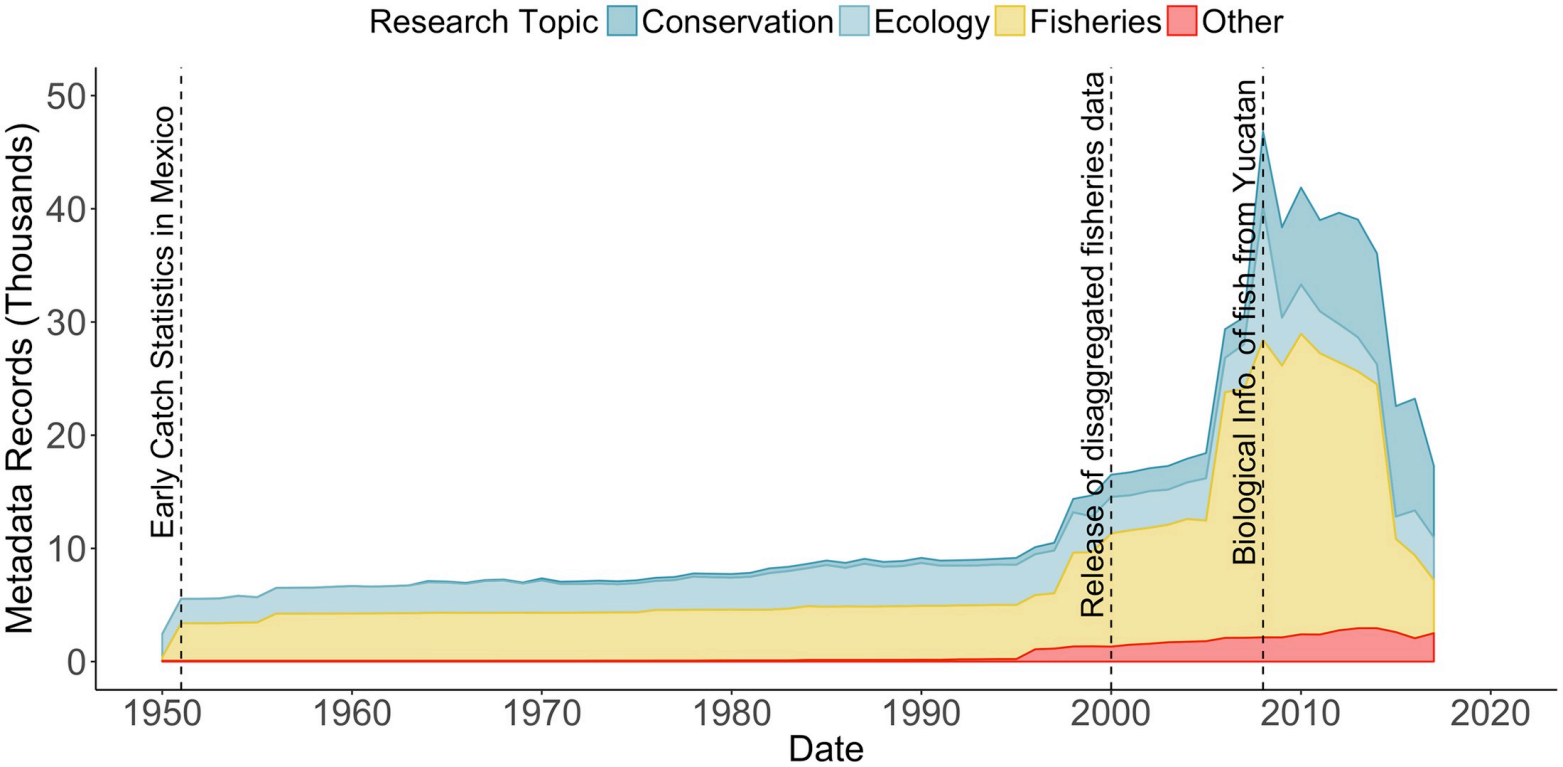
Yearly metadata records by major research category. Results shown from year 1950 onward. See Fig 1B for categories included within “Other”.

Analyzing metadata collection years shed light on historical research trends as reflected in available data (Fig 4). The first metadata records dated back to data collected in 1791 (plankton records), and data on ecology were historically well represented with several collection events through time. Most fishery records begin in the early 1950s, expanding later as local research increased, with a remarkable increase in records on conservation topics around the first decade of the 21st century. Our analysis also shows a downward trend in total records starting around 2010 and an abrupt drop around 2015 (Fig 4). We believe this trend from 2015 to date are probably due to the delay in gathering and preparing information before it is made available.

There are 24,083 subjects (taxa target of the data colelction) represented in the metadatabase. Most single-subject records (97%) represented taxa (e.g. *Octopus maya*, or *Epinephelus* spp.) and only 3% was identified with common names such as “Octopus” or “Mangrove”. Assessments not differentiated by a single subject are grouped under “Multiple species” and comprised only 3% of all records. While the list of species in the metadata was quite large, data availability was uneven: 3.7% of subjects with most metadata records comprise 52.29% of all records. Subjects with the most amount of records were Carcharhinidae shark species *Carcharhinus porosus* and *C*. *falciformis* with 1,200 records each, followed by *C*. *limbatus* with almost 1,000 records.

**Fig 5:**
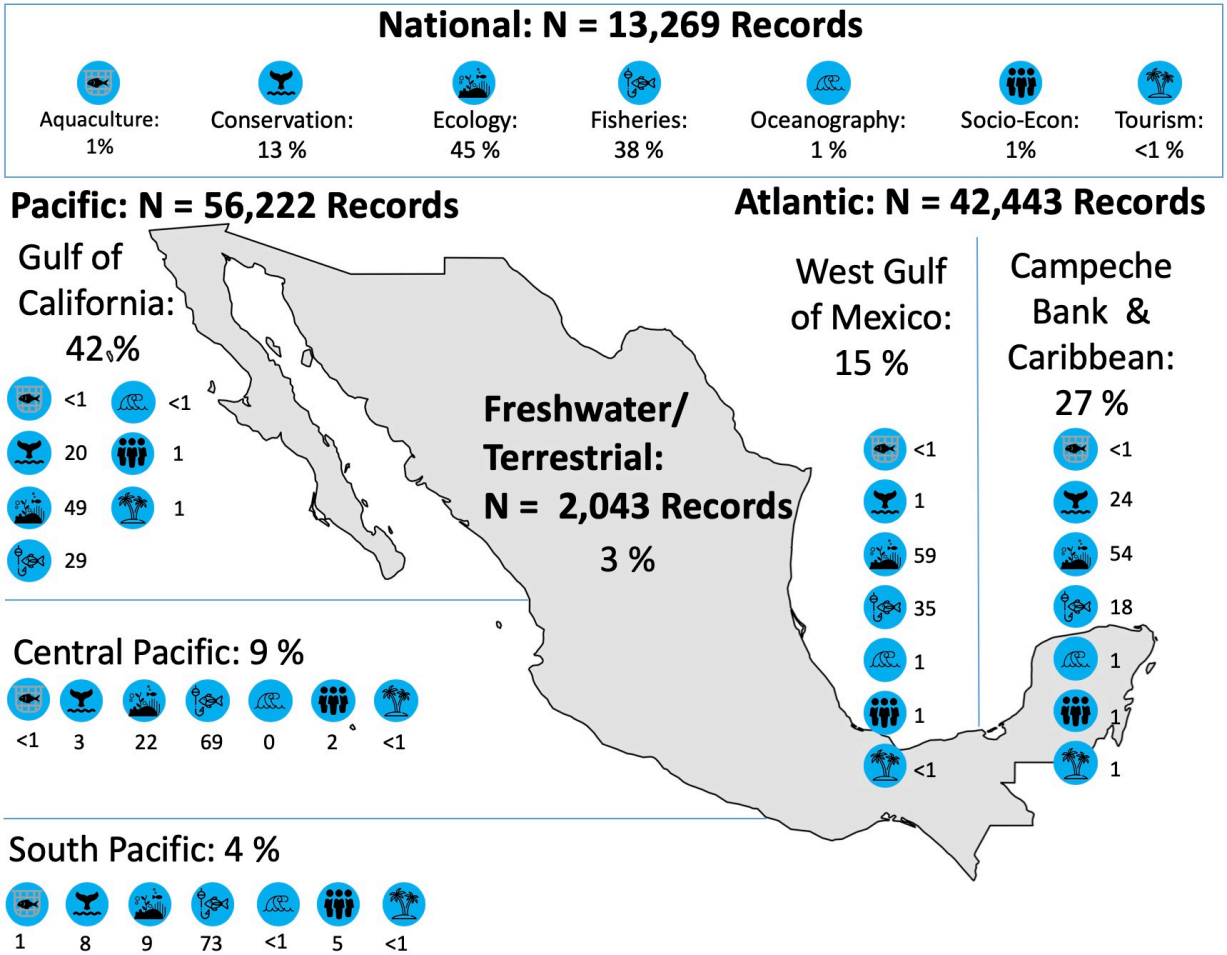
Geographic location of metadata according to sub-regions and research category. All values are in percentage except those that say “Record”; numbers within regions may not add to 100% due to exclusion of “other” types of research. Icons from Freepik (https://www.freepik.com) downloaded from https://www.flaticon.com on 07/12/2018. Map reprinted from Natural Earth (http://naturalearthdata.com).

There were significant differences in the distribution of metadata between oceans ($X^{2}=93114$, d.f. = 6, *p* < 0.001) with more data from the Pacific (49% of records, though mostly in specific zones) than the Atlantic (37%); the additional 14% of records were reported at the national level. Regional differences were significant ($X^{2}=63175$, d.f. = 3, *p* < 0.001), with more records available for the Gulf of California and Northwest Mexican Pacific (42% of all records, and 77% of records within the Pacific), followed by the Campeche Bank and Caribbean region (27%) (Fig 5).

**Fig 6:**
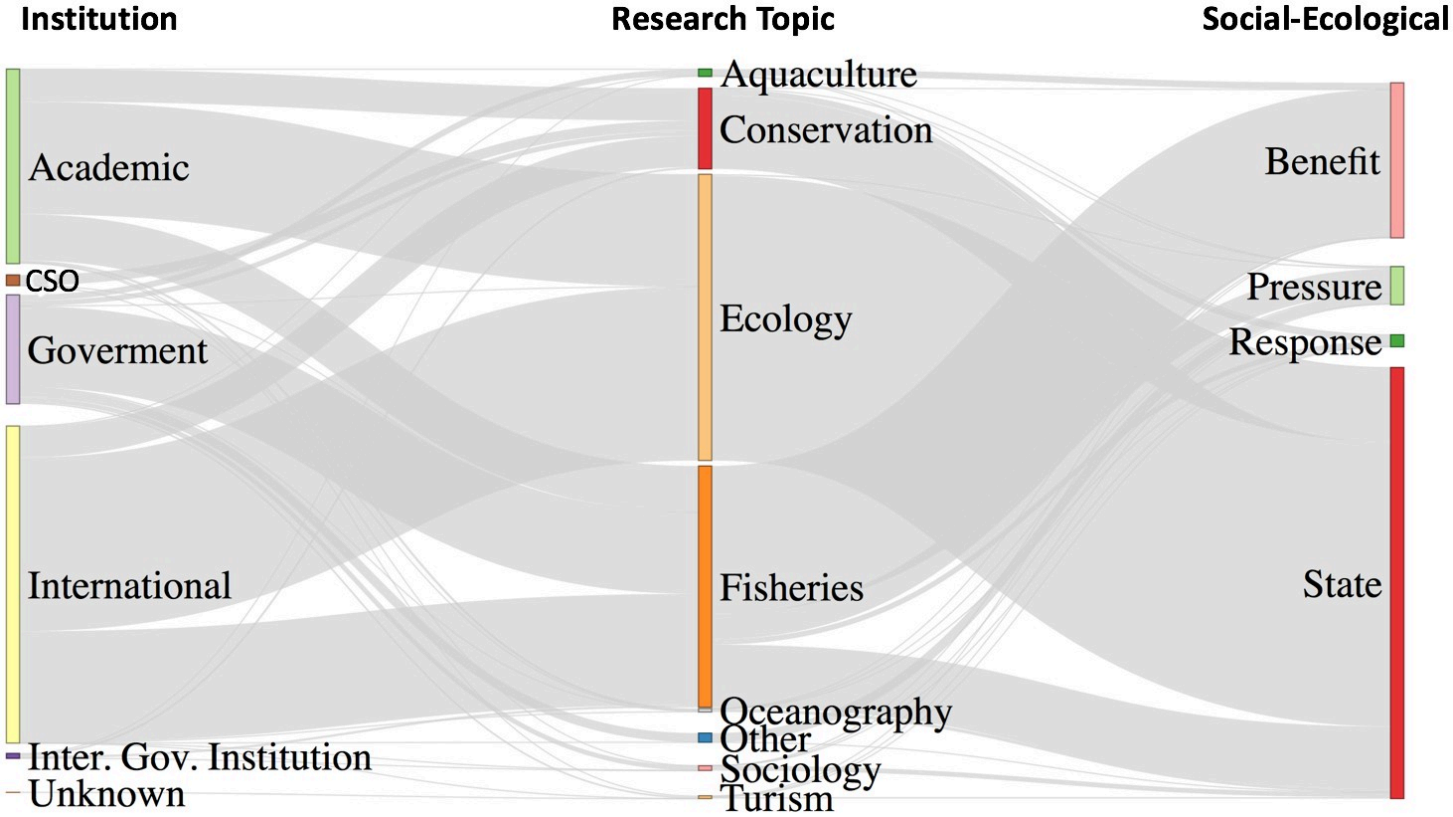
Characterization of institutions that host data, research field, and social-ecological interaction indicators. Thickness of grey connections represents the number of metadata records.

For Mexico, most data generated in the academic sector was catalogued as *State* (e.g. species listings), with governmental information mainly reporting *Benefits* (e.g. tourism expenditures). Government agencies also provided information regarding *Pressures* on ecosystems, such as fishing subsidies, number of active fishing vessels, and so on. Finally, records from non-governmental institutions (national and international) mainly relate to the state of natural resources and social benefits such as employment (Fig 6). Sparse information about conservation topics was available regarding social benefits, and comparatively smaller amount of fisheries or aquaculture research addresses pressures versus benefits. Information regarding *Responses* is underrepresented in the metadatabase for all research fields.

## Discussion

Metadatabase analysis of Mexico ocean data helped us to understand the availability of multi-disciplinary ocean-related information and data, identification of status and trends of research and available information, as well as knowledge gaps to support marine-related policy-making. Particularly, building a metadatabase of marine research allows for an overall evaluation of research and data trends that is useful for decision making [13]. Our analysis of collected metadata revealed Mexico’s long-term history of marine research with substantial ecological and fisheries-related data mainly on academic and government research institutions, respectively. However, we identified a need to incorporate and/or invest in long term ecological monitoring, other aspects of fisheries landings and other topics such as conservation and oceanography. Examples of these efforts can be found in initiatives like FMCN-Monitoreo Noroeste and the Long Term Ecological Research Network (LTER-Mex), databases [61]. Such efforts will certainly support policy progress towards sustainability goals such as the Convention on Biological Diversity Archi targets [14]. We also identified a skewed regional distribution of data towards the Gulf of California and North Pacific and almost non existing in other areas of the Pacific. This result highlights that there is either a data gap in the regions other than the Gulf of California and North Pacific, or that available data are less assessable in these poorly represented areas. The results from this study may help raise the awareness that resources to support more marine research and/or enhancing collaboration in knowledge exchange between institutions are needed in the regions.

General trends in available data over time, as reflected in metadata, can be attributed to major national and international initiatives. Increases in available Mexican data in the 1950s stemmed from the request of the United Nations’ Food and Agriculture Organization for developing countries to compile and report data on the state of national fisheries [29,62]. Worldwide, this increase in data availability enabled further research initiatives to complement policy-relevant information at local, regional and global scales (e.g. Sea Around Us [63], the Ocean Health Index [5], and Too Big To Ignore—Information System on Small-scale Fisheries (TBTI-ISSF) [64].

Government efforts since the early 2000s have drastically improved fisheries data availability [62], including the annual CONAPESCA fishery yearbooks (in database format) [65] and the Open Data portal [39]. Ecological and conservation metadata also increased during this period, mainly through academic and CSO monitoring programs; particularly large repositories include the UNAM-UAY for the Yucatan Peninsula, and COBI in the Caribbean, both of which have open data policies (Fig 3). The systematic study of the marine social-ecological systems by CSOs in the Gulf of California was prompted after federal law allowing CSOs to be established in early 2000s [66]. The first decade was dedicated to organization, but consequently the first programs on fisheries and biodiversity were established once CSOs, government agencies, and academics developed a more formal relationship. These partnerships resulted in the availability of abundant information which in later years has informed specific conservation initiatives [67], research initiatives and their scientific outputs [68,69]. Decreasing trends in available data in recent years may be explained by various factors, and most likely a lag between data collection and availability (due to processing or publication times) [13], and funding constraints for data collection on specific topics that may historically have provided more data [62,70,71].

It is interesting that many overall trends found in the Mexico metadata are comparable to research available for Canada, that used a similar metadatabase approach with almost identical categories that help in comparisons [13]. For example, around 60% of all records in the Canada metadata corresponded to fisheries, and fisheries are indeed the largest contributor to research on use in Mexico (Figs 3 and 4), with ecology being the second-highest and highest contributor to records for Canada and Mexico, respectively [13]. There is also a strong prevalence towards research on single species (e.g. catch, life-history traits and presence/absence data), with these representing around 70% of records for Canada [13] and over 90% in Mexico. However, research on ecosystems themselves has been increasing in both countries since the late 1990s, a likely reflection of the cementing of the ecosystem-based approach as a key aspect of management of marine resources around this time [72,73], and also a relatively extensive research capacity in Mexico despite it being a developing nation. However, information on themes beyond fisheries or resource use itself are currently under-represented in the MDB, and particularly highlights a need for increased attention to research on the human dimensions of marine systems to inform integrated ocean assessments and support inclusive decision-making processes. This is not a limitation specific to research in North America, as comparable metadatabase projects from Australia [17] and the Canary Islands [18] show very-well documented and extensive information on species and ecosystems but little on the social characteristics of marine resource users.

Although the long history of ecological data collection in Mexican waters produced several species catalogues from marine invertebrates to fishes and mammals [74], there is a substantial difference in metadata consistency between commercial and non-commercial species. Ecological data tend to be sporadic observation records, as most projects do not maintain long term monitoring series due to restrictive costs or time-bound funding restrictions [13]. In contrast fisheries data collected have more consistent time-series, with more long-term monitoring records as compared to other ecological data, and for that reason represented the highest number of data points in the metadata (Fig 3). Thus, a commercially important fishery species in the metadatabase can have more than 50 years of catch data while non-commercial species often have a single observation record over the same time period. The overwhelming relative amount of information on fished species is understandable and not unique to Mexico [75], but ecosystem-based approaches to management require a much wider array of data, at the very least to adequately account for impacts from fisheries [76]. Furthermore, research not specifically related to current human uses is crucial to evaluate interactions, externalities and potential future responses to system shocks.

Regional differences in data availability reflected underlying research trends, but also differences in the regional capacity of institutions, and ecosystem and social-economic patterns [77]. The Gulf of California region, among the most biodiverse areas of the world [78] and of paramount importance for Mexican fisheries, has become a hub for academic research and conservation and fisheries-related initiatives. These research institutions provide the infrastructure to subsequently generate large amounts of data [77]. In contrast, the south-central Pacific of Mexico and the western Gulf of Mexico have far fewer fisheries research centers, CSOs, and education institutions than the rest of the country [77]. Unsurprisingly, these areas are also the least represented in the metadatabase and should be prioritized in future metadata collection.

In the Gulf of Mexico, the catastrophic environmental and economic impact caused by the Deepwater Horizon well blowout in 2010 [79] highlighted the limited ecological data available to evaluate impacts and prompted increased scientific research supported in Mexico by federal agencies. Data produced from these new research are mostly not available yet due to ongoing litigation between governments, fishing and tourism associations, and oil producers, but this will eventually provide important information for the region. In addition, the development of important inter-institutional initiatives such as The Gulf of Mexico Research Consortium (CIGoM) based at the CICESE, CINVESTAV [80], and the Harte Research Institute [81], and the project of Marine Biodiversity of the South of the Gulf of Mexico led by the Marine Biodiversity Lab (BDMY) [82] will help lay the foundations for a marine observatory in the region.

We highlight three main lessons learned from the creation of the MDB and further metadata analysis that should be taken into account for future efforts. First, despite the benefits of data sharing [10,16], a range of institutional barriers often hinder the exchange of data (and even metadata) among stakeholders [27]. These barriers include a lack of incentives to publish datasets (in terms of academic citations), unwillingness of data sharing by owners fearing to be scooped out of the project [83], and technological limitation in maintaining and sharing large datasets for long time [27]. A change in these systems can provide a better work environment, foster collaboration and boost interdisciplinary marine research. For example, Mexico’s educational system requires that most science students (from bachelors to PhD) produce theses including new datasets. However, such documents are not always digitalized (and rarely for older theses) and are difficult to find without previous knowledge of their existence; this type of information could easily be integrated into the metadata structure described here, opening up a significant opportunity to appreciate and link the work of young researchers throughout the country [77]. Moreover, recent legal changes mandate that all scientific and technological information derived from research and educational programs fully or partially funded with public resources must be open access. To achieve this, CONACYT was charged with the creation of a National Repository, itself fed by institutional repositories, that would store, maintain, and preserve scientific information [84].

Second, Mexico’s higher education network extends to more than 500 research institutions across 32 states [85], and government agencies such as INAPESCA have offices throughout the country [77], this is undoubtedly good in terms of research capacity but makes it very difficult to exchange information or engage in discussions. This can be beneficial as decentralized researchers can better address local issues [68], but it also requires innovative strategies for collecting information (e.g. in the form of metadata), eliminating bureaucratic barriers to information sharing and facilitating collaborations across regions and institutions.

Finally, the internet is a vast dynamic and growing space, with new datasets and repositories becoming available at a rapid pace (sometimes daily). The current project partnered with CONABIO, a government agency specifically tasked with collecting, maintaining, and making data available, to produce a dynamic metadatabase that would continue to gather and share information through a user-friendly portal. Aside from this technical and strategic capacity to make scientific information widely available, CONABIO is the largest repository for natural science research and information on fields beyond, but related to, marine ecosystems. The incorporation of the marine metadatabase can therefore become an important addition to wider knowledge, particularly given that the management of marine living resources requires an integration with atmospheric and ocean physics, freshwater basins, and land-based processes with direct and indirect feedbacks. Similarly, future metadata collection should further increase efforts to identify data related to emerging Ocean Economy sectors aside from fisheries (e.g. wind energy, blue carbon, ecotourism, bioprospecting), which are included here but will likely be the focus of more research in the future.

The process of creating a multidisciplinary metadatabase framework, compiling metadata, and exemplifying potential analyses with preliminary results provides general trends of data availability and facilitates cross-disciplinary collaboration. In addition, transforming the MDB in an open access online platform, that is user-friendly and edditable improves the longevity of the metadatabse, and improves access and utilization of information to better inform policy and management strategies for complex systems [12,86].

## Conclusion

The metadatabase approach developed here is intended as a cost- and time-effective way to identify information and research trends, strengths, and gaps, as well as a channel for researchers to communicate their science and engage in new collaborations. Incorporating a wide array of institutions and researchers, and making the best use of emerging technologies, can certainly improve on this type of metadatabase approach, both in Mexico and elsewhere. We consider that this effort can and should be repeated in other regions and countries. The ultimate goal of a metadatabase is to facilitate a multidisciplinary approach to informing social, environmental, and economic sustainability policies that are inclusive and effective across time and scale. The most updated version of the metadatabase of marine research in Mexico can be found at https://www.infoceanos.conabio.gob.mx.

## Supporting information

S1 Table List of all 29 metadata categories in the metadatabase. (CSV)

S2 Table List of places where data was collected. This list includes host institutions where we held (or participated in) workshops, meetings or presentations related to the metadata repository and compilation. Events organized by the authors were open invitations and Attendees shows the estimated number of people at each session.
(CSV)

S3 Table List of abbreviations used in the current document. (XLSX)
